# The future of sleep health: a data-driven revolution in sleep science and medicine

**DOI:** 10.1038/s41746-020-0244-4

**Published:** 2020-03-23

**Authors:** Ignacio Perez-Pozuelo, Bing Zhai, Joao Palotti, Raghvendra Mall, Michaël Aupetit, Juan M. Garcia-Gomez, Shahrad Taheri, Yu Guan, Luis Fernandez-Luque

**Affiliations:** 1 Department of Medicine, University of Cambridge, Cambridge, UK; 2 The Alan Turing Institute, London, UK; 3 Open Lab, University of Newcastle, Newcastle, UK; 4 Qatar Computing Research Institute, HBKU, Doha, Qatar; 5 CSAIL, Massachusetts Institute of Technology, Cambridge, MA USA; 6 BDSLab, Instituto Universitario de Tecnologias de la Informacion y Comunicaciones-ITACA, Universitat Politecnica de Valencia, Valencia, Spain; 7 Department of Medicine and Clinical Research Core, Weill Cornell Medicine - Qatar, Qatar Foundation, Doha, Qatar

**Keywords:** Preventive medicine, Biomedical engineering, Diagnostic markers, Sleep, Predictive markers

## Abstract

In recent years, there has been a significant expansion in the development and use of multi-modal sensors and technologies to monitor physical activity, sleep and circadian rhythms. These developments make accurate sleep monitoring at scale a possibility for the first time. Vast amounts of multi-sensor data are being generated with potential applications ranging from large-scale epidemiological research linking sleep patterns to disease, to wellness applications, including the sleep coaching of individuals with chronic conditions. However, in order to realise the full potential of these technologies for individuals, medicine and research, several significant challenges must be overcome. There are important outstanding questions regarding performance evaluation, as well as data storage, curation, processing, integration, modelling and interpretation. Here, we leverage expertise across neuroscience, clinical medicine, bioengineering, electrical engineering, epidemiology, computer science, mHealth and human–computer interaction to discuss the digitisation of sleep from a inter-disciplinary perspective. We introduce the state-of-the-art in sleep-monitoring technologies, and discuss the opportunities and challenges from data acquisition to the eventual application of insights in clinical and consumer settings. Further, we explore the strengths and limitations of current and emerging sensing methods with a particular focus on novel data-driven technologies, such as Artificial Intelligence.

## Introduction

Sleep is a crucial biological process, and has long been recognised as an essential determinant of human health and performance. Whilst not all of sleep’s functions are fully understood, it is known to restore energy, promote healing, interact with the immune system and impact upon both brain function and behaviour^1,2^. Even transient changes in sleep patterns, such as acute sleep deprivation, can impair judgement and cognitive performance, whilst long-term aberrations have been linked to disease development^3,4^. Global trends in sleep suggest a decrease on average sleep duration^5–8^. Given these trends and the implications of sleep for health and well-being, better characterisation of sleep characteristics represents a public health priority^9–11^.

Sleep is known to be regulated by three main factors: circadian rhythms, sleep–wake homoeostasis and cognitive-behavioural influences^1^. With regards to behavioural determinants, poor sleep quality^12^ (as defined by the National Sleep Foundation’s recommendations based on total sleep time, sleep latency, wake after sleep onset, number of awakenings >5 min and sleep efficiency) has been associated with stress, anxiety, smoking, sugary drink consumption, workplace pressures, financial concerns, regularity of working hours, physical activity, sleep regularity and commuting times^9,13^. Indeed longitudinal research has linked changes in physical activity to changes in the severity of sleep-disordered breathing and, hence, disturbed sleep^14^. Furthermore, dietary patterns have shown associations with sleep quality^15^. It is now understood that the associations between diet, physical activity and sleep are bidirectional. Thus, poor sleep, high levels of inactivity and a poor diet comprise inter-related public health priorities^16^. The mental and physical impairments associated with a single night of poor sleep can outweigh those caused by an equivalent lack of exercise or food^17^.

Sleep loss affects every major system in the human body. Chronic changes in sleep have been associated with a plethora of serious medical problems from obesity and diabetes to neuropsychiatric disorders^13,18,19^. For example, chronic insomnia is associated with both incident cardiovascular disease and all-cause mortality^4,20^. A 2011 meta-analysis of prospective studies, which included 470,000 individuals, explored the association between sleep duration and cardiovascular disease^21^. Relative to those who slept between 7 and 8 h per night, those who slept less than 6 h exhibited a 48% increase in the incidence of coronary heart disease and 15% increase in the incidence of stroke, whilst those who slept greater than 8 to 9 h exhibited a 38% increase in coronary heart disease, a 65% increase in stroke and a 45% overall increase in cardiovascular disease^21^. Other large epidemiological studies have also reported associations between sleep and cardio-metabolic disease, including reports studying the effects of shift-work^22–25^. Short sleep duration has further been associated with incident diabetes and weight gain, as well as impaired appetite control^18,26^. Shortened sleep and poor sleep quality have also been identified as risk factors for cognitive decline, neurodegenerative disease, mood changes and depression, as well as other neuropsychiatric conditions^27–30^. There is also mounting evidence linking sleep to both immune function^31^ and cancer^32,33^. In a seminal study published in 2002, Spiegel et al. demonstrated an association between sleep deprivation and a muted immune response to flu vaccination^34^.

Besides its ramifications for the health of individuals, sleep has macro-level economic implications. A recent study estimated the annual economic cost of poor sleep to the Australian population at $45.2 billion, comprising direct healthcare costs, the cost of associated health conditions, reduced productivity, accidents and informal care^10^. Moreover, in a 2016 report, RAND Corp quantified that the combined cost of insufficient sleep across five OECD countries (Canada, USA, UK, Germany and Japan) exceeds $600 billion a year^9^.

Following mounting evidence of the role of sleep in well-being, its relationship with disease and mortality and its economic impact, there has been increased interest in measuring sleep characteristics. This has led to an expansion in the development and use of sleep-related technology. In particular, recent developments in digital technologies designed to improve the measurement and characterisation of sleep have demonstrated particular potential. These advances facilitate the objective and unobtrusive measurement of sleep characteristics in large, free-living populations at scale, facilitating well-powered epidemiological investigations designed to explore the relationships between sleep and disease^35^. Furthermore, these developments are set to have clinical implications for the monitoring and diagnosis of sleep disorders and, ultimately, could be used for the modulation of sleep^36^.

**Fig. 1:**
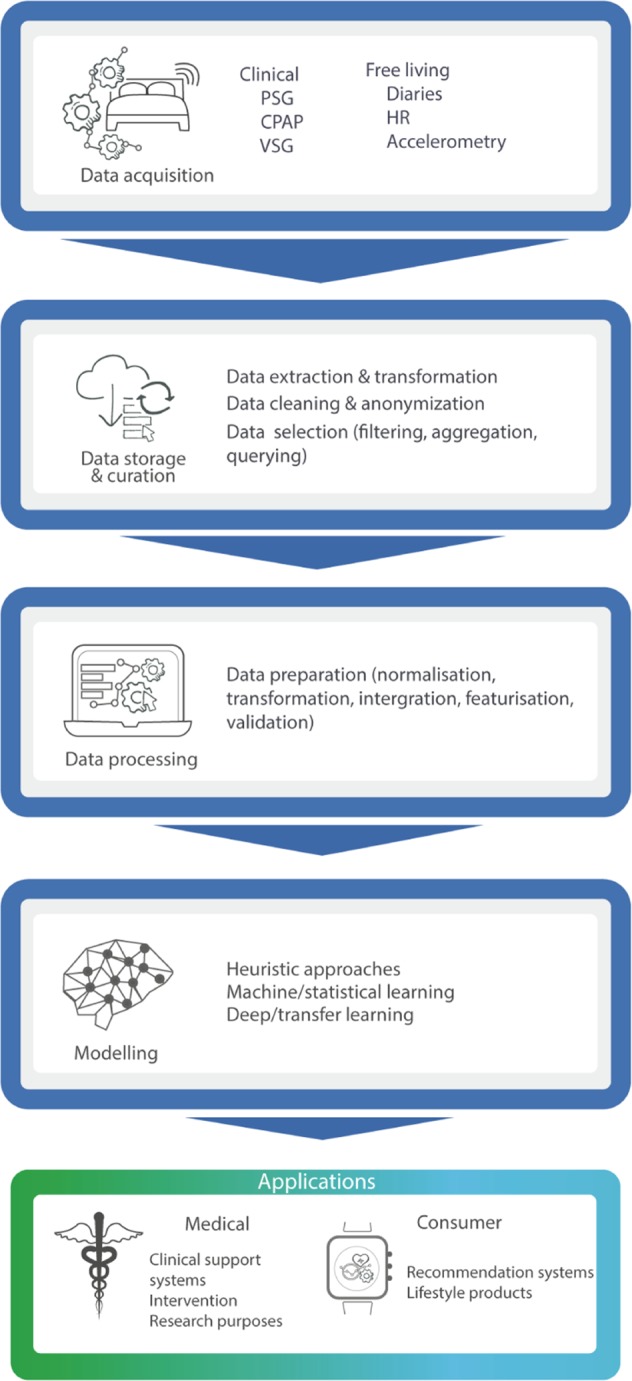
The digital sleep framework covers the path of sleep data from its acquisition to when its insights are used for medical or consumer applications. The framework begins with the acquisition of sleep-related data. This can be done using a variety of sensors, ranging from polysomnography to bed sensors. This data is then stored and curated, a step that comprises privacy-aware storage, cleaning, filtering and anonymisation. Once that data has been appropriately treated, the processing step takes place whereby data is transformed and integrated based on the end-model. For example it may undergo different transformations like normalization or featurization. The next step entails modeling, which can consist of simple heuristic methods, statistical learning or deep learning methods, for example. Finally, the resulting model can be deployed for a variety either medical or consumer applications.

The core contribution of this paper is to discuss the implications of digital technologies for the study, monitoring and modulation of sleep. To aid this discussion, we introduce a five-step Digital Sleep Framework, which comprises the complete process from sleep data acquisition to end-user applications of insights. Figure 1 depicts the framework. This paper is structured around the framework’s five steps: data acquisition; data storage and curation; data processing; modelling and applications. Finally, we discuss the biggest challenges and opportunities in this field followed by conclusions based on our findings.

## Sleep data acquisition

Sensors have been used to study sleep for decades. Traditionally, polysomnography (PSG), paired with clinical evaluation, has been the gold-standard and de-facto technique to study sleep in clinical and laboratory settings, as well as to diagnose a subset of sleep disorders^37^. However, in recent years, industry and academia have invested heavily in the development of smaller, less obstructive and more portable devices for the continuous monitoring of sleep^38^. This is motivated by a desire to enable data acquisition in larger participant groups over more extended periods and in a more natural setting by decreasing both the cost of monitoring and the burden to participants. However, challenges remain in data acquisition, including the provision of ubiquitous, less obtrusive and stigmatising long-term acquisition mechanisms. Moreover, long-term patient monitoring usually suffers from missing periods that may mislead the estimations of health markers. Here, we discuss the current state-of-the-art of sleep data acquisition in clinical and free-living settings, including an overview of traditional and novel approaches and their strengths and weaknesses.

### Traditional sleep monitoring and monitoring in laboratory settings

Since the 1960s, polysomnography (PSG) has been used in clinical settings to monitor sleep through a battery of simultaneous, complementary sensors^39^. These sensors typically allow for the measurement of (1) brain activity through electroencephalogram (EEG), (2) airflow, (3) breathing effort and rate, (4) blood oxygen levels, (5) body position, (6) eye movement, (7) electrical activity of muscles and (8) heart rate. Traditionally, PSG requires participants to sleep in a laboratory setting. The results are then scored by an expert who has received training on how to interpret these signals. Ambulatory PSG is an alternative modality which often uses a reduced number of sensors and allows monitoring to occur at home, outside of the laboratory. This facilitates the monitoring of patients with disorders that may not be easy to evaluate in a laboratory setting^40^.

To-date, PSG remains the gold-standard for sleep measurement. However, the technology is limited in its use as it remains impractical for long-term home use. This precludes its use in long-term sleep monitoring or sleep in free-living settings beyond the laboratory. Furthermore, PSG is expensive, time-consuming and requires trained technicians to administer and interpret. As a result, the scalability of this technique for large-scale population-based studies is very limited, particularly when the aim is to assess typical sleep patterns in free-living, naturalistic conditions. Whilst ambulatory PSG provides a partial solution to some of the issues, it remains both expensive and burdensome.

Another conventional method used in clinical settings to evaluate sleep is Videosomnography (VSG). VSG encompasses a range of video-based methods used to record a person as they sleep. These video recordings are subsequently used score sleep behaviours. VSG has been typically paired with PSG in clinical settings to study sleep disorders. However, recent advances in telemedicine have made the use of home VSG increasingly possible. Although VSG is typically scored by experts in a time-consuming manner, advances in signal processing and AI have led to the new possibility of automatically scored VSG^41^. However, VSG presents similar scalability issues as PSG. It is costly and, at present, requires expert monitoring and scoring.

### Sleep monitoring outside the laboratory

To understand the role of sleep in health and disease, sleep must be monitored in a free-living environment and in a non-obtrusive way to ensure the sleep captured is as representative of typical sleep as possible. As such, low-cost, wearable sleep detection systems are a promising tool to study sleep architectures in free-living individuals at a population level. At present, there are several options for the monitoring of sleep outside the laboratory. These comprise actigraphy, heart rate sensing and other wearable technologies. Multiple published works have demonstrated that a single modality sensor representation, such as heart rate alone, is not sufficient to accurately complete sophisticated sleep stage classification^42^. The availability and range of digital technologies for the measurement of sleep has significantly expanded in the last decade. Both consumer and medical grade devices across a variety of fields (wearable, remote sensing, mobile health, clinical grade) have become more sophisticated and affordable. Nevertheless, comparing performance across different platforms and methods remains a challenge, and few methods have been validated against gold-standard PSG or undergone systematic reliability assessment^43^.

### Traditional free-living sleep sensing and measurement approaches: actigraphy and accelerometry

Actigraphy and accelerometry are non-invasive methods to monitor human activity and rest cycles. They have been used to describe physical activity levels in large-scale populations^44^, and can also be used to monitor sleep. These methods offer an affordable, scalable alternative to PSG to monitor sleep–wake cycles, and have now been recognised by the American Academy of Sleep Medicine as a valid method for the assessment of sleep^45^. Recent advances in AI and larger studies in conjunction with PSG have resulted in the refinement of the method^46^. However, three key limitations of actigraphy and accelerometry remain. These are (1) the lack of validation studies for the different consumer-grade devices, (2) lack of standardisation of approaches for human-activity recognition and (3) the lack of assessment techniques for daytime sleeping. Nowadays, wearable sensors are often used in combination with other minimally invasive sensors (such as heart rate monitors, miniaturised ECG, pulse-oximetry, blood pressure monitors, galvanic skin conduction, light sensors, gyroscopes, barometric altimeters and GPS trackers). Sleep can also be monitored through a combination of wrist actigraphy, hip sensors, smartphone sensors and under-mattress sensors^47^. Nevertheless, this increased availability of sensors also results in a greater challenges when optimising the match between the end-application and the sensor used^43^. A description of actigraphy specific sleep metrics is provided in the Supplementary Material.

Data on sleep can be also obtained from devices to treat sleep apnoea, such as Continuous Positive Air Pressure (CPAP). For example, Aggarwal et al.^48^ showed that CPAP can be used to classify and track sleep metrics, which could be used to monitor the response of CPAP therapy in sleep apnoea patients.

### Emerging sleep-sensing technologies

**Fig. 2:**
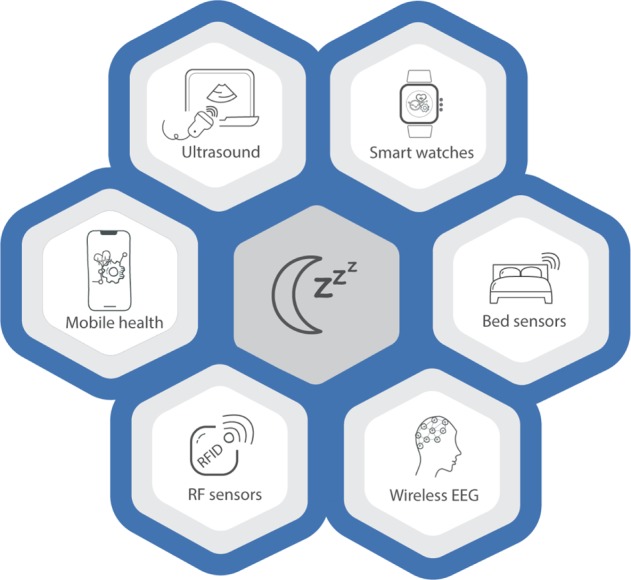
Emerging sleep-sensing technologies. Emerging sleep technologies range from non-contact methods like RF sensors to miniaturized, wireless or in-ear EEGs.

The fundamental aim of ubiquitous computing in sleep tracking is to achieve miniaturisation of sensors and non-intrusive sensing that can pervasively monitor physiological signals related to sleep activities. Embedding different types of ambient sensors into objects that we interact daily is more attractive than using multiple redundant sensors collecting homogeneous information. Embedded devices, such as bed sensors, have been developed to track different sleep-related metrics, such as sleep time, breathing, snoring, heart rate, body and room temperature or humidity levels^49–51^. Whilst these sensors are interesting and potentially valuable for clinical and epidemiological research, as well as wellness and sleep education, very little is known about how their performance against gold-standard measures and more research is required to evaluate their usability. Some have emerged in recent years but remain at an early stage of development (e.g., WiFi and radio-signal approaches), whilst others have been around for longer (e.g., smartwatches). They are depicted in Fig. 2 and discussed below. Some of the potential techniques to unobtrusively measure sleep through the acquisition of physiological signals include the following:

#### Bed sensors

Bed sensors may be defined as any sensor that sits on the bed and can be used for monitoring physiological processes. Body movements, breathing and even cardiac activities can be detected by the volume change of the pneumatic underneath an individual whilst they are lying in bed^52–54^. For instance, using micro-bend fibre optic sensors underneath the mattress allows for monitoring of breathing and body movement activities that can be then used to extrapolate some valuable sleep metrics^55^. Similarly, fibre-optic based systems have allowed not only for the analysis of different motion types but also for the introduction of retroactive feedback based on those movements^56^. Unobtrusive sleep monitoring using bed sensors (either on the mattress or the bed frame) usually entails monitoring of movement and also respiration rate and occasionally heart rate. Several companies, including Apple (Beddit), Nokia and Withings, have released new sensor accessories that can be attached to a person’s mattress or bed frame and often interact with a separate mobile application or dashboard. Nevertheless, a range of determinants can influence the performance of these methods, from postural differences to inter-subject variability in BMI and pre-existing clinical conditions^54^.

#### Consumer-graded wireless EEG and reduced-array EEG

EEG is an integral part of PSG and is also used in a variety of neuropsychiatric tests and applications. Conventional EEG requires expert set-up and can be burdensome, uncomfortable and is not portable. Wireless EEGs have gained traction in recent years, with several established companies, as well as start-ups, launching products. Their performance for sleep monitoring has been compared with conventional EEG that is part of PSG and has demonstrated strong results^57,58^. Furthermore, Koley et al.^59^ showed that automatic scoring using ensemble models on a single-channel EEG could yield agreement rates of 0.87 when compared with expert scoring of the same signal. Whilst this study was conducted in a clinical environment, and hence lacks the recording conditions required for free-living validation, together these investigations show that the results of conventional EEG can be approximated by simpler devices that may be able to be scored automatically.

Similarly, several miniaturised EEG devices have shown promising results with regards to their ability to classify sleep stages^60,61^. In ear, EEG is a modality that has shown promise in recent years, for instance, Mikkelsen et al.^62^ compared in-ear mobile EEG analysed through machine learning-based automated scoring to conventional, manual scored PSG and commercial-grade actigraphy showed promising results, although also constrained to a laboratory environment. A 2019 study showed that automatic sleep stage prediction based on a single in-ear sensor demonstrated a 74% agreement with the hypnogram generated from full PSG, which is promising but still requires further work for it to be at a clinical standard of performance ($\approx$90% agreement)^63^. These devices are particularly interesting given their potential for free-living application. They also have the advantage of conserving much of the granularity and information that a conventional PSG-based EEG would offer in non-laboratory set-ups^64^.

Although the performance of these wireless, miniaturised and in-ear EEG devices is promising, more extensive studies are required to determine the feasibility for use in population science and in a free-living environment as well as for applied sleep research studies.

#### Smartwatches and fitness trackers

A plethora of wearable smartwatches and activity bands have been developed to infer sleep. These devices often derive their metrics using a combination of movement signals (accelerometry, as explored in previous sections) and heart rate and heart rate variability. Henriksen et al.^65^ assessed the validation or reliability of some of the most common brands on the measurement of physical activity and sleep (Fitbit, Garmin, Misfit, Apple, Polar, Samsung, Withings and Mio).

#### Mobile phone sensing

Mobile phones offer a wide range of sensors, such as gyroscopes, microphones and accelerometers, that can be used to monitor sleep patterns^66^. For instance, iSleep, developed by Hao et al.^67^, leverages a smartphone’s built-in microphone to detect events that happened during sleep, such as body movement, cough, and snoring by processing the acoustic signals. The software achieves accuracy of over 90% for event classification (snoring, cough, sleep) under different environmental conditions. An important limitation of the system is that the high-rate microphone sampling represents a significant source of energy (and battery) consumption.

Several other sleep applications can be found on the different app stores these days. Sleep cycle is among the most popular ones, using both accelerometry and the built-in microphone to track sleep and provide personalised alarm clocks, waking up the users at ideal timings (during light sleep)^68^.

#### Ultrasound sensors

Ultrasound sensors can be used to detect body movement and breathing patterns during sleep^54,69,70^. These sensors provide information regarding the frequency and type of body movement through the Doppler technique. This technique mirrors that used in conventional radar systems and allows the retrieval of parameters related to breathing rate, heart rate and body motion. The method has been shown to detect physical movements with an 86% recall rate and error rates of <10%^71^. The most pressing limitations of this method are, however, the fine-tuning required based on the type of targeted body and the sensitivity to small movements^72^.

#### WiFi and radio-signal approaches

In the past decade, high frequency and sub-millimetre wavelength radio technologies have demonstrated the ability to capture physiological signals without body contact. The principle is to send a low-energy radio wave towards an individual who is in bed and then to detect the signal bounced back from the body. Through signal processing, it is possible to extract biological information, such as breathing patterns, heart rate and full-body motion from these findings^71,73–75^. These biological signals can be used to determine sleep stages as shown by Zhao et al.^76^, as well as to monitor insomnia^77^. The main challenge with this approach is that the signal is subject to a lot of ‘noise’ and the information related to sleep needs to be extracted. Moreover, the measurement conditions are also strongly dependent on the individuals being monitored. In particular, the signal reflects all objects in the bedroom and is affected by the sleeping position of the individual^78^.

**Fig. 3:**
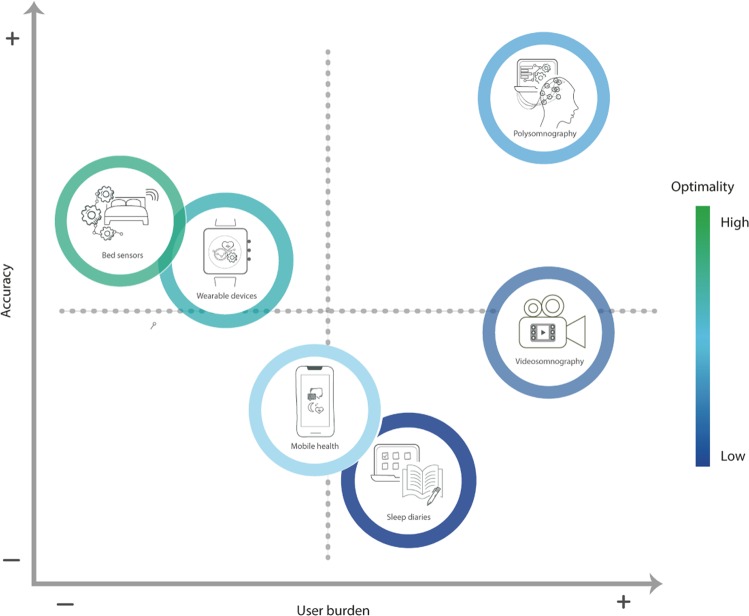
Selected methods for the measurement of sleep and their accuracy and usability trade-off. This chart plots the accuracy of sleep-sensing methods at infering sleep-related metrics against their ease of use. For example, while polysomnography is considered the “gold-standard” technique to measure sleep, it is cumbersome and expensive.

Some of the methods described in this section are, in general, more accurate or more usable than others. Figure 3 shows a scheme of the accuracy versus usability trade-off for the main methods described in this section.

**Fig. 4:**
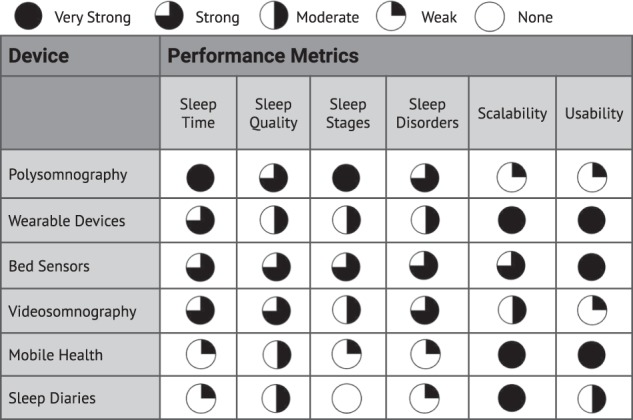
Holistic evaluation of sleep-monitoring methods. Some methods, such as PSG, are accurate but inappropriate for use in daily sleep monitoring, as they require professional set up and are intrusive. Other methods, such as bed sensors, are unobtrusive but more prone to noise than PSG.

Data collected from different modalities representing diverse physiological information may have varying predictive power and noise topology as explored in Fig. 4. However, different modalities and the information they collect may be highly complementary and, in practice, aggregating sleep data from various sources may make models more robust and tolerant both to noise and missing data. Such complementary fusion protocols have been shown to significantly improve the classification performance of sleep stages^79,80^.

## Sleep data storage and curation

Regardless of its intended end-use, all the data collected using the methods and sensors previously discussed requires appropriate storage, curation and processing prior to analysis. Until the turn of the century, analogue PSG systems, limited to analogue amplifiers and paper tracings, were common practice for storing sleep information. However, with the development of digital recording systems, these types of analogue recordings have become outdated, as different challenges have emerged for handling data from new digital sleep technologies. For example, in the era of digital medicine, systems often require real-time storage and processing of data collected as part the so-called Internet of Things (IoT)^81^ and Big Data Analytics^82^. IoT links all sorts of connected devices into comprehensive networks of inter-correlated computing intelligence without with need for human input. With regards to sleep, the integration of IoT technology has several challenges. These include data storage, management and exchange across different devices and sensors, alongside privacy, security and data access concerns.

**Fig. 5:**
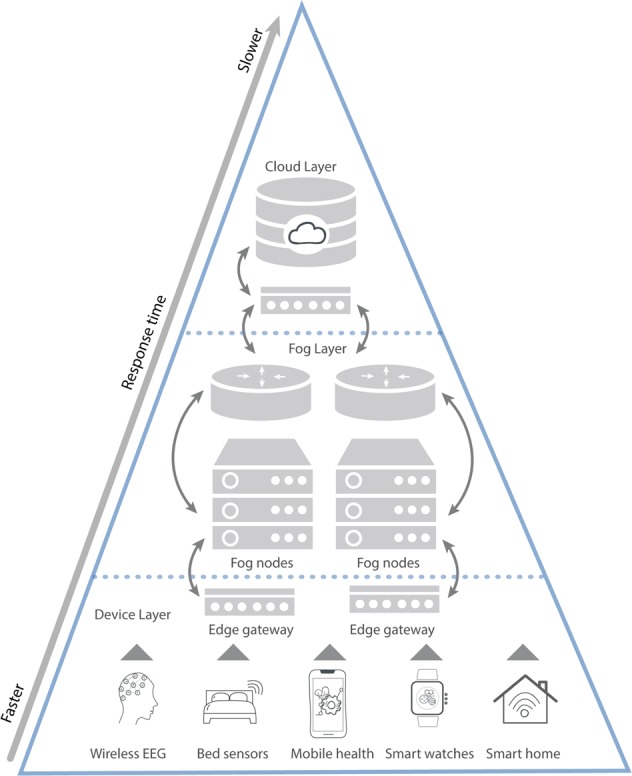
Overview of cloud computing-based sleep data acquisition and storage. This illustration provides an overview of the process starting with device layer (which includes fast, real-time processing and data visualisation, embedded systems, gateways and micro data storage), followed by the fog layer (which includes local networks, virtualisation, data analysis and reduction) and finally cloud layer (which consists of data centres and big data storage and processing).

Cloud computing integration with IoT is gaining traction in healthcare, and is being used for digital sleep applications. For instance, three-layered architectures composed of (1) an IoT layer sensor acquisition/data compilation; (2) a fog computing layer for event processing and (3) a cloud layer for data management and Big Data Analytics have been proposed for sleep monitoring use cases that integrate several sensors^83^. In Fig. 5, an overview of data acquisition and the movement of information from sensors to the cloud is explored. The remainder of this section discusses the fog computing and cloud storage layer more fully.

### Fog computing layer

Fog computing entails data analysis on edge devices, which enables real-time data processing, reducing costs and also improving data privacy. Fog computing is commonly deemed mini-cloud computing, as it performs all the processing locally. The fog computing layer abstracts the heterogeneity of the incoming data formats, communication technologies and protocols from the sleep-sensor IoT layer. Platforms, such as Smart IoT Gateway, have emerged as solutions to communicate with all the heterogeneous IoT sensors potentially deployed in home environments and perform local processing before transmitting the data to the cloud layer^84^. Fog computing seeks to achieve a seamless continuum of computing services connecting the cloud to the devices (IoT). This contrasts to edge computing which isolates and keeps the computing at the ‘network edges’^85^ and facilitates the aggregation of multi-modal physiological data from different devices and sensors that are then processed locally (e.g., processing data directly on an IoT Gateway). This architecture can provide near real-time decision-making to support sleep monitoring and intervention.

Following the receipt of signals from the devices, pre-processing at the fog computing layer includes three main operations: (1) the fusion of signals provided by different IoT sensors; (2) detection of periods containing missed data and (3) imputation of missed data. When sleep sensor signals contain missing data, it is usually because the user did not wear or was not in contact with the sensors. However, functional errors can also occur. For example, smartwatches may run out of battery or memory and may fail to communicate with the user’s smartphone. Missing data can be detected by various algorithms, including through simply thresholding the smoothed signal.

Besides data pre-processing, the fog computing layer also enables the inter-operability of heterogeneous sources of the data. Inter-operability is a key function of Smart IoT Gateways. It allows for communication and integration of devices, which are operated on different protocols and use different technologies. Furthermore, the Gateways facilitate the sharing of information and the driving of actuators or components that meet the required needs of the system^86^. For instance, it can be used to detect sleep apnoea events and activate motors designed to change the users’ body position or to play sounds or music during particular sleep stages^87^.

### Cloud storage layer

Cloud computing architectures include servers, networking, software, databases and data analysis over the internet which enable fast deployment, flexibility and economies of scale. Cloud computing is often considered the centralised paradigm, while the fog computing layer previously described would be a decentralised paradigm. Nevertheless, as explained in Fig. 5, they can effectively work together.

Sensor data integrity is paramount for successful application and analysis in digital medicine and requires appropriate data storage in order to be realised^88^. Relational databases can be limited when storing and analysing semi-structured data obtained from multi-modal sleep-sensing technologies. Hence, current trends to store and query digital sleep data are based on Not Only SQL (NoSQL) databases such as MongoDB, Cassandra, HBase or CouchDB, which allow for better representation of heterogeneous data structures and batch data. Moreover, several of these NoSQL databases provide connectors to cluster-computing frameworks, such as Apache Spark, Storm, Flink and Hadoop, enabling Big Data Analytics. These Apache products are a good fit for both batch processing and stream processing via in-memory computation and processing optimisation^89^. Resilient Distributed Dataset (RDD) allows Apache Spark to simultaneously store data on memory and write to storage media based on pre-defined criteria from the real-time data stream. Hadoop allows for batch processing and the use of MapReduce algorithms to analyse data stored in Hadoop Distributed File System (HDFS). HDFS can handle petabyte level data analysis, which can be used to provide in-depth statistical analysis of clinical sleep data and can also be used in large population epidemiology studies.

## Data pre-processing

Before sleep data can be used for modelling, it must be pre-processed. As discussed in the preceding sections, there is a growing trend towards the integration of sleep data from various sensors. As such, there is a preponderance of unstructured multi-modal time-series data with substantial noise. For example, different equipment brands and models may be equipped with different quality of sensors, amplifiers and electrodes that result in different noise topology as a result of their unique materials and manufacturing process. Data measurement, processing and storage may also differ between sensors. For example, depending on the application and device, it might store RR interval, instead of raw ECG. Hence, data need to be cleaned and filtered, removing artefacts that differ depending on the modality employed before any feature extraction or modelling can take place.

Depending on the nature of the data, several pre-processing approaches can be applied. Smoothing and de-noising can remove unwanted spikes, trends and outliers from a signal^90^. For example, polynomial de-trending methods can remove continuous quadratic or linear trends that may be caused by impedance changes on the skin. Similarly, Hampel filtering can remove unwanted spikes from sinusoidal signals. Noise arising from other sources should also be be considered. This may include power line interference, thermal-based resistive changes or contact conductive artefacts. These noises can be filtered by applying various bandpass filters. The ultimate objective of de-noising is to ensure that the noise is subject to a specific distribution, such as a Gaussian distribution, as far as possible.

Beyond de-noising and smoothing, re-sampling and standardising can be used to improve data integrity and consistency in the pre-processing stages. Linear or higher-order interpolation can be used to fill missing or corrupted data, as well as for data scaling, through methods such as linear scale-transformation^91^. These methods can suppress the noise levels and variability in the signal and transform the data into a pre-defined range without altering its distribution. Data standardisation, such as min–max standardisation and *z*-score standardisation, can suppress noise levels and variability in the signal and transform the signal such that it approximates a normal distribution.

## Artificial intelligence-based sleep modelling

Once sleep data has been pre-processed, data modelling can be commenced for different applications. Today, many of these modelling and application tasks are based on AI, which entails the use of algorithms and techniques that mimic human cognitive functions, reasoning and problem-solving skills and have brought a paradigm shift to digital medicine. Indeed, the influence of AI in medicine is growing rapidly and is being exploited in a variety of fields from clinical medicine to population studies^92^. In essence, the application of AI in medicine aims to aid clinical decision-making through analysing complex medical data. The insights generated can then be used in diagnosis, treatment, the prediction of clinical scenarios and to aid scientific discovery^93^. Increasingly, AI is changing research methodology and facilitating the personalisation of medicine through its advancements^92^.

With regards to sleep science, the impact of AI is multifaceted. First, it can aid clinicians in making sleep disorder diagnoses^94^. This is achieved by translating collected sensor data into pre-defined knowledge (e.g., class label), providing an inexpensive and objective alternative to manual sleep stage scoring^95^. Similarly, through its automated analysis capabilities, AI can provide wellness and lifestyle recommendations based on the interpretation of data collected from wearable devices and mobile apps^96,97^, enable clinicians and researchers to track changes in sleep patterns from people’s homes^98^ or interact with smart-home set-ups to provide better quality sleep through the adjustment of lights and temperature in rooms^99^. Here, we discuss methods of AI-based sleep modelling.

### AI applied to sleep science

Traditional AI systems were rule-based, requiring the programming of pre-conceived rule sets and demonstrating limited flexibility. By contrast, machine learning (ML) provides a more flexible alternative to data modelling, especially when applied to the raw unstructured signals. In plain terms, ML aims to train, learn and optimise a mathematical model which can transform or map the collected (complex) signals into comprehensible knowledge.

Usually, ML approaches, which include logistic regression, support vector machines and random forest, tend to use structured data as input. This makes feature engineering or feature extraction a standard procedure before model training. Feature engineering can be achieved in various forms. For example, given a sliding window (from the raw time-series data), statistical features such as mean, standard deviation, energy, entropy and so on, or time–frequency features such as wavelet/Fourier transform coefficients can be extracted and used as input for the traditional ML models. Moreover, in some applications, domain experts can also design features based on their understanding of the signal in certain fields. Compared with the raw signals, the engineered features tend to be low-dimensional with information redundancy suppressed, making the model training tasks more effective.

From a ML perspective, the most common tasks for sleep research are the classification of sleep–wake cycles and stages as well as the derivation of sleep–wake metrics. Although heuristic approaches and some traditional ML approaches have demonstrated reasonable performance in some tasks, the feature engineering process tends to be time-consuming, and may require domain knowledge in some circumstances, making the whole system design an expensive process. On the other hand, the new methodologies offer more flexibility in sleep modelling. For example, deep-learning methods can be used to perform end-to-end training, which directly maps the raw signal into the target labels. It is a pure data-driven process, and latent patterns can be automatically learned without the feature engineering process.

In Table 1, we highlight several popular ML models that can be applied to different sensing modalities.

**Table 1:** Sleep classification techniques across different sleep-sensing modalities.

Technique	Technique variations	PSG/EEG	Wearable sensing
Statistical	Latent dirichlet allocation	^167^	
	Support vector machines	^168^	^169^
	Hidden Markov model	^170^	^171^
	Quadratic	^172^	
	Bayesian	^173^	
	Logistic regression	^174^	^107^
Instance base	K-nearest neighbours	^108^	^109^
Decision tree	Decision tree	^175^	^35,176^
Ensemble model	Adaboost	^177^	
	Bagging	^178^	
	Random forest	^179^	^180,181^
	XGBoost	^182^	
Clustering	K-means classifier	^183^	
	Spectral clustering GMM	^184^	
ANN and DNN	Convolutional NN	^185^	^76,107^
	Recurrent NN (LSTMs, GRUs)	^186^	^107,187,188^
Others/heuristic	Fuzzy classifier	^189^	
	Wavelet methods	^190^	
	Sadeh		^45^
	Sazonov		^191^
	Oakley		^192^
	Cole-Kripke		^193^
	Webster		^194^
	ADAS		^195^
	Scripps clinic		^196^

### Conventional sleep classification methods

Following the American Academy of Sleep Medicine (AASM) guidelines, traditional sleep scoring in neurophysiology laboratories assigns 1 of 6 labels to each 30-s epoch. These are as follows: (1) awake; (2) rapid eye movement sleep (REM); (3) non-rapid eye movement (Non-REM); (4) sleep stage 1 (N1); (5) sleep stage 2 (N2) and (6) sleep stage 3 (N3). This task is performed manually by trained sleep technicians based upon data generated through PSG. Sleep stages themselves are associated with physiological changes that are useful for the diagnosis and assessment of specific sleep disorders such narcolepsy^100^. For example, respiratory monitoring in PSG facilitates the detection of sleep-disordered breathing, such as obstructive sleep apnoea. In this disorder, abnormal breathing events are less severe in N3 than N1 sleep due to the change in central control of breathing, and more severe again during REM given upper airway muscle tone reduction^101^.

**Fig. 6:**
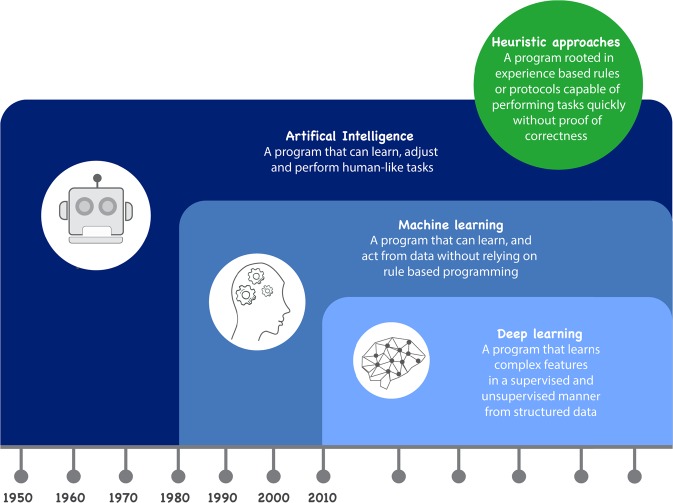
Sleep classification algorithms can be based on heuristic approaches or Artificial Intelligence. We describe machine learning/statistical learning approaches and deep-learning approaches within AI.

Manual sleep scoring suffers from several drawbacks. It is time-consuming, subject to biases, inconsistent, expensive and must be done offline. Rosenberg et al. reported that the average inter-scorer reliability for sleep stage scoring was approximately 83%^102^. This estimate is similar to that reported in other studies^103^. By contrast, the use of AI and automated sleep stage classification algorithms represents a fast, non-subjective, inexpensive and scalable alternative to this traditional sleep-scoring approach. Aside from issues of reliability, it can take 1–2 h for an expert to score a night of clinical PSG recordings^104^, whilst the automated system can finish the same task in seconds. Thus, multiple approaches and methods have been used to distinguish sleep from wake automatically as well as to characterise specific sleep stages. In broad terms, sleep classification algorithms fall into different categories, but these categories can be closely inter-related, as shown in Fig. 6. The different categories comprise traditional algorithms and both ML and deep-learning approaches. These are elaborated below.

*Traditional algorithms* for scoring of sleep from either PSG or actigraphy signals tend to be based on heuristic approaches^105^. These heuristic approaches are themselves based on prior knowledge of the sensing modality and sleep physiology. In actigraphy, the use of the magnitude feature as a proxy of movement for sleep/wake classification provides one example. This approach offers quick solutions with fast implementation and also tends to be biased by the programmer’s understanding and interpretation of the problem and to perform differently depending on the population, in which they are applied. For example, the algorithm developed for a nocturnal sleep pattern may not suitable for non-nocturnal sleep. Penzel et al. provided an in depth review of some of these approaches in clinical settings, offering a quantitative analysis of their performance and requirements^106^. Palotti et al. evaluated the performance of some of the most common approaches, including statistical ML, on actigraphy data^107^.

*Machine learning and deep learning* approaches have gained traction in recent years for the task of classifying sleep–wake cycles and sleep stages in multi-modal sensor data^108,109^. With the availability of raw actigraphy signals, several deep-learning techniques such as convolutional neural networks^110^ and recurrent neural networks^111^ have been used to exploit the temporal nature of this unstructured data to distinguish the sleep–wake cycles^107^ robustly and understand the role of activity in sleep-related disorders^112^. Whilst the evaluation of most traditional and ML algorithms are performed using standard quality metrics such as accuracy, precision and recall per class, it is also important to measure clinically relevant metrics such as waking after sleep onset (WASO) and sleep efficiency^107^. By optimising clinical metrics, ML methods enable the physicians to make informed, clinically relevant decisions. Adequate performance defined by quality metrics varies depending on the task intended. For instance some sleep disorders may not require high levels of granularity for their diagnosis whereas interventions that aim to boost deep sleep ought to rely on accurate granular classifications of sleep stages.

Table 1 provides a holistic overview of the most common classification methods based on the modality used (PSG/EEG, wearable device (accelerometry/actigraphy), others (heart rate/PPG/etc)). References are provided for methods by modality in the appropriate cells. It is important to note that different methods are ought to be used based on the objective at hand. For instance, deep-learning methods often provide better performance than traditional statistical learning methods, but require large computational power and lack the interpretability that other models offer^107^. Performance and model evaluation is discussed in further detail on the Supplementary Material.

### Emerging approaches for sleep classification

There are a plethora of methods available for the predictive modelling of sleep-related problems, as mentioned in previous sections. However, several outstanding questions remain regarding their application. Issues such as model sustainability, handling the heterogeneity of the data and variability in the demographics, behaviour and lifestyle of the population and generalisation to unseen data, need to be investigated more comprehensively. Below, we highlight some of the emerging technological solutions for the handling of these issues.

#### Model sustainability

An important consideration is that the majority of existing ML models perform a task (such as sleep–wake classification and sleep-related disorder prediction) by learning from an underlying distribution of data. However, in real-world conditions, the data generated from participants can change over time due to age, lifestyle changes, new sensing modalities, the progression of sleep/health disorders or other changes. An imperative question then is how to make the trained model sustainable in response to changing domains. Life-long learning might be the first step to address some of these challenges. This would facilitate sustainability by allowing the model to evolve over time^113^.

#### Personalised sleep classification

One of the major challenges that AI encounters when facing sleep classification tasks is inter-subject differences. That is, the intra-class variability (e.g., differences in length of REM sleep between participants) can be too large to be captured by the trained model, making inference process prone to errors. By contrast, by taking personal information into account, a human analyst can address this problem easily. In the case of PSG scoring, a skilled neurophysiologist may consider demographic characteristics (such as age and gender) and adjust their scores accordingly. Despite advances in AI methods for sleep classification across different sensing modalities, most of the current models do not adapt to individual characteristics. There may exist large inter-subject variation and the trained model (on the population-level data) may not be the optimised one for certain individuals.

In the future, personalisation could be a useful approach to improving the performance of the AI-based sleep modelling systems, improving the performance of algorithms^114,115^. It has been suggested that personalisation may be especially useful when using data from noisy modalities, such as wearable devices^116^. Model personalising has been successfully applied in other fields, such as mood recognition^117^ and seizure detection^118^. However, it remains relatively untapped in sleep science. Recent works have shown that transfer learning could be used to realise personalisation. For example, based on EEG modality deep neural networks were trained on a large population, followed by a fine-tuning process at the subject-level^116^. The results suggested that substantial performance gain can be achieved^116^.

The process of personalisation can also be applied in the aforementioned distributed networking environment. Federated learning proposes a distributed way of updating a centralised model by aggregating each patient’s local updates into a central server^119^. The distributed update framework not only provides a model parameter update mechanism but also creates a personalised predictive model by feeding individual data to a global model in the localised updating process.

#### Generalised sleep classification

Another way of improving the performance of AI-based systems is to reduce the effect of the contextual information for better generalisation^120^. Based on adversarial training process, some of the most recent works performed subject-invariant learning, which makes the system less sensitive to personal and environmental factors^112,121^. Similarly, by undergoing an adversarial training procedure, temporal dependencies can be learned. These then transfer well to new subjects and different environments in sleep classification tasks^76^. Pillay et al. used EEG data in combination with a generative modelling process to obtain agreement between the labels estimated and clinician’s labels for automatic four stage sleep classification in infants^67^. In general, by learning the representative features that are less sensitive to contextual factors and thus robust in various (complex) sleep classification tasks (such as diagnosing sleep apnoea or insomnia), these approaches aim to increase the generalisation capabilities for better performance. This is an alternative to the aforementioned personalisation approaches.

## Data-driven sleep applications

**Fig. 7:**
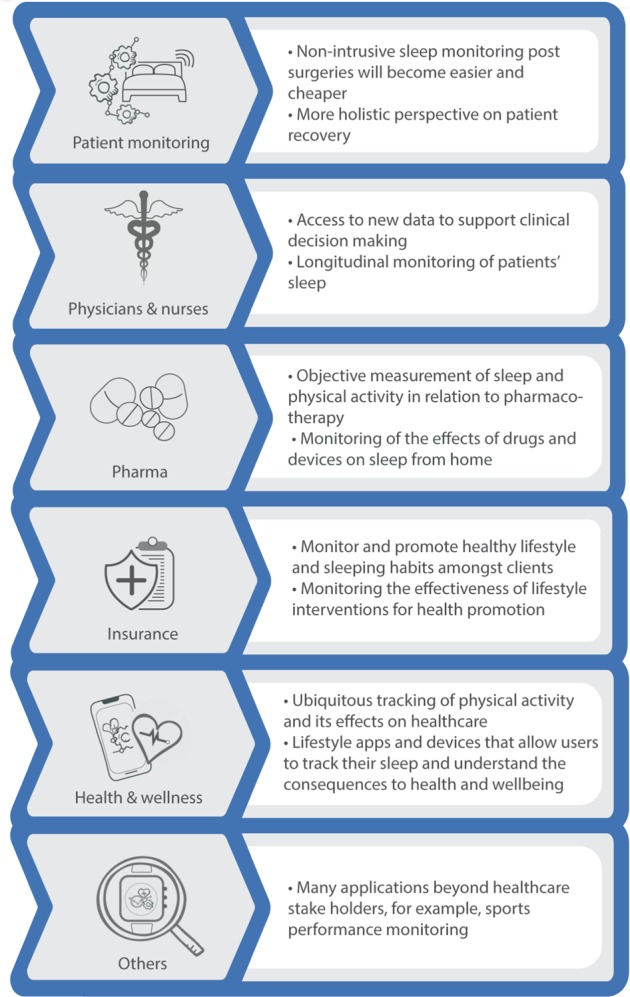
Key areas of impact for sleep health. Emerging sleep health technologies will have an impact on patient monitoring, clinical care, insurance, the pharmaceutical industry and health and wellness applications, as well as other impacts including on digital therapeutics and sports performance.

There is a wide and growing range of commercial, health and clinical situations for which data-driven sleep applications are being used. In hospitals, sleep medicine units have traditionally used PSG and more recently actigraphy/accelerometry, for the diagnosis and monitoring of sleep disorders^122^. One of the main challenges in sleep medicine is the increasing incidence of sleep disorders, which in turn leads to higher demand on sleep labs to provide diagnoses. Consequently, software for sleep medicine is being gradually upgraded to include automated sleep-metric calculations and seamless integration of sleep data sources, such as sleep questionnaires. These upgrades have the potential to compliment Electronic Health Records, enabling healthcare practitioners to better manage their patients sleep disorders^123^. Figure 7 gives a brief overview of key areas that will be affected by the impact of sleep technologies and newly generated sleep data.

### Sleep data in health and disease

As discussed, disturbed sleep has been linked to reductions in quality of life and to a higher risk of a plethora of chronic conditions^124,125^. Thus, it is of vital importance for digital self-management and monitoring solutions to include tools that allow accurate monitoring and assessment of sleep quality. Aside from its direct role in ill-health, poor sleep quality can worsen the symptoms of many serious and chronic conditions, including cancer and multiple sclerosis^126–128^. Moreover, pharmacological treatments may in turn worsen sleep disorders as side effects. For example, smoking-cessation drugs and some treatments for cancer have been shown to reduce patients’ sleep quality^126,129^. Due to the complexity of the relationship between sleep and health, there is a need for the design of digital intervention methods to address the unique requirements of sleep within long-term or chronic conditions. There are early examples of mHealth interventions to improve sleep quality on people with cancer and diabetes, amongst others^130–132^.

Amongst otherwise healthy individuals, there is also an increasing interest in mobile and wearable applications for health and wellness^97^. Ultimately, it has been proposed that such technologies could be used to direct personalised sleep health recommendations to individual users^36^. Furthermore, other consumer sleep technologies have gained traction in recent years, and although they still need appropriate clinical evaluation, they could enhance patient–clinician interaction and sleep self-management^133^. Some of the most common commercial and familiar technologies, such as Fitbit or SleepAsAndroid, offer monitoring and tracking sleep quality. In addition, new sensing technologies, such as those discussed in the sleep data acquisition section, are gaining traction and devices like Beddit have attracted investments from large technology companies^134^. These monitoring technologies are complemented by applications aimed at improving quality of sleep by supporting a more suited wake timing using approaches such as smart lighting or smart alarms that only ring when the user is in light sleep. Despite their growing popularity, at present, most of these consumer-oriented technologies lack validation and their underlying models change frequently^97,133^. This fast growing industry needs to be matched by multidisciplinary scientific efforts that evaluate the performance, usability and value proposition of new sleep technologies.

When exploring the impact of the digitisation of sleep on wellness and health promotion, it is also important to mention occupational health applications. Often sleep disorders are the result of lifestyle factors including, for example, prolonged screen time before bed. The resulting poor quality of sleep can feedback to that lifestyle, by reducing productivity. Consequently, corporate and health insurance wellness programmes are starting to offer incentives and personalised coaching to clients and employees, with some initiatives directly promoting sleep quality at the workplace^135^. For instance, FirstBeat provides a solution for companies that comprises personalised sleep and physical activity monitoring for employees combined with personal face-to-face coaching with the aim of increasing employee health and employment satisfaction^136^. However, these technologies can also be exploited, as in West Virginia prior to the teacher’s strike, where declining to wear a fitness tracker and meet a certain step count resulted in a $500 penalty annually for their healthcare payments.

### Data visualisation and visual analytics

Data visualisation, in general terms, is the graphic representation of data. Abstract data are processed such that they can be represented using visual objects (e.g., points, lines, bars, etc.) ease of interpretation and better understanding. Visualisation relies on human’s high throughput visual perception channel, and the ability to connect data representations to human knowledge and expertise which are not encoded directly in the data^137^.

Visualising health-related data goes back to the days of paper charts and maps. Since the rise of internet and mobile application, digital displays are ubiquitous and people are now widely educated to read standard graphics representing data. Typically, activity data are presented based on the time component, which is usually visualised using line charts, where the horizontal $x$-axis is time. Raw signal visualisation is mainly meaningful for domain experts trained and experienced to interpret complex patterns. Specific patterns can be automatically detected or highlighted on the chart, for instance, when activity levels go above or below a threshold^138^. Projection techniques are also a popular means of reducing the dimension of high-dimensional data for better visualisation and knowledge generation^139,140^.

Sleep data visualisation is only meaningful if the resulting visualised data make sense to the end-user, which can be challenging for non-expert users of wearable technology. SleepExplorer is an example of visualisation research aimed at understanding how users can benefit from visualising their own personal sleep data^141^. SleepExplorer organises a flux of sleep data into sleep structure, guides sleep-tracking activities and highlights connections between sleep and other related factors such as napping, coffee and alcohol intake, as well as mood. Recent studies have analysed behavioural change resulting from techniques implemented in activity trackers and their visualisation, but few studies are focused on sleep^142^. Ravichandran et al. conducted a study of user’s experience and understanding of sleep metrics provided by sleep sensing devices. Their findings suggest that visual feedback may be helpful to users^143^.

However, several challenges remain in regards to sleep data visualisation. (1) Scalability: for large-scale historical health data, visualisation requires adapting to large time scales (from minute-level to year-level information) and displaying meaningful data summaries to the user or primary care practitioner. (2) Heterogeneity: the data collected from different devices varies greatly from, for example, GPS location or glucose levels to pictures of food or phone-screen time. This poses a challenge for the visualisation of personal data for patients and for the healthcare professional^144,145^. (3) Usability: sleep data visualisation should be tailored to end-users and their specific needs^146^.

## Challenges and opportunities

With advances in technology, the volume of physiological and clinical data resources available to biomedical research is expanding^147^. This includes open-source data from Electronic Health Records, medical image repositories, genomic archives and massive person-generated data from wearable technologies^147–149^. Recently, sleep data repositories, such as Sleepdata.org, have been created to advance the field^150^. These repositories include multi-modal sleep data (from clinical-grade PSG to actigraphy and questionnaires)^150^, and are being used to create ML benchmarks^107^. These developments are crucial for the creation of generalised ML models that can be applied reliably to clinical and commercial settings to further our understanding of the role of sleep in well-being and disease.

Sleep-related technologies are not only useful for monitoring but may also be used to aid intervention. For example, the portability and pervasive use of mobile phones makes them an attractive option for the delivery of interventions and several studies have already shown promising results when using mobile phone platforms for sleep interventions. These interventions include, but are not limited to sleep advice for behavioural change^151,152^, optimised alarms based on sleep stage^153^ and sleep tracking and feedback^96,154^. Furthermore, new sleep technologies may be able to complement or augment current clinical-grade diagnostic tools for sleep disorders. A 2017 review by Shin provides an in depth overview of this area of research as well as the strengths and limitations of the current efforts^155^.

Despite the potential of technologies and open resources, challenges must be overcome if their potential is to be realised. Their heterogeneity, variability (both between sources and over time) and data quality is, at present, a strong barrier to efficient data reuse. Appropriate analysis also remains a challenge. To overcome this, temporal and source variability of signal repositories must be characterised^156,157^ and common representation spaces should be defined to exploit shared latent information among data distributions. Indeed, appropriately representing data and metadata originating from different sensors (type, make, version, etc) is critical in order to later harmonise and integrate data from disparate sources as well as for sensor data fusion. Models should adapt their inferences from different data sources and at different points in time.

In addition to data handling and analysis challenges, new sensing technologies require systematic validation^158^. These validation requirements vary based on the end-use of the technology, and must be held to higher standards if they are to be used in clinical settings^158^. On a population level, there is a wide and growing interest from the general public in wellness mobile and wearable applications, which in many cases are related to sleep and inform people’s lifestyle decisions and understanding of their health^143^. Nowadays, there are hundreds of sleep applications and a plethora of wearable devices that claim to track sleep quality^159^. However, most of those devices have little or no information regarding their reliability and validity, the testing they underwent or how the data is acquired (i.e., sampling rates, pre-processing, etc) and processed^160,161^. As such, individuals could become concerned or reassured about their sleep based on unreliable data. Further, concerns have been raised about the performance of these devices in populations with chronic conditions and mobility problems^162^. Whilst this has, to-date, mainly been confined to concern regarding the tracking steps and physical activity, these devices must be tested in a range of populations, in particular, those with sleep problems. Massive usage of consumer-grade sleep tools may also increase individual’s health concerns and have a ripple effect on overstretched healthcare systems^133^.

Lack of reliability and validity testing also poses several obstacles to the use of data-driven applications in sleep medicine and research. As explained in a 2016 editorial by Wilbanks and Topol^163^, the lack of transparency in these technologies limits researchers’ capabilities to study any potential bias due to the lack of information on the characteristics of the cohorts. Further, this lack of transparency makes it more difficult for researchers and clinicians to use these devices and mobile applications. Despite some initiatives, such as Apple HealthKit or C3-PRO^164^, which aim to facilitate data sharing across platforms, these data tend to be highly summarised and in a post-processed state. Summary-level data is not always appropriate for use in academic research or some AI applications, as the processing steps are often not described.

To generate maximum benefit to the end-user or other stakeholders (e.g., hospitals, researchers, public health officials, regulators, industry), there is an increasing need for a safe and effective clinical biomarker ecosystem with algorithmic transparency, inter-operable components and sensors and open interfaces that allow for high integrity measurement systems^165^. This will allow for the verification and validation of digital biomarkers for sleep health.

Finally, there are data-privacy concerns. Sleep tracking mobile applications and wearable technologies often collect information such as movement, GPS location and sound, which could have potential applications beyond the tracking of sleep. These privacy concerns may be mitigated through the deployment of data-processing functions on the user’s mobile equipment, without requiring server processing^166^. Similarly, an alternative is to empower users to decide what data they want to send to the server^166^.

## Conclusions

The impact that sleep has on human health is undeniable. Recent advances in sensing technology, big data analytics and AI allow for truly ubiquitous and unobtrusive monitoring of sleep and circadian rhythms. However, challenges remain to realisation of the benefits of this monitoring for individuals, research and clinicans. Here, we introduced the Digital Sleep Framework, a framework outlining the steps required from the multi-modal acquisition of sleep-related data through to its clinical and commercial application and exploring all aspects of this chain. As the number and scope of sleep monitoring technologies continues to grow and the diversity of digital sleep solutions and applications continues to multiply, the need for careful, risk-based product validation has become increasingly important. The heterogeneity of sensors used for the monitoring of sleep–wake cycles and circadian rhythms poses a unique set of challenges for modelling and interpretability. Hence, the identification and standardisation of robust, reproducible digital sleep biomarkers is of paramount importance. Modelling based on these signals must be as free as possible from conscious and unconscious bias and the development of algorithms must be transparent and readily available for all stakeholders.

The digitisation of sleep is likely to have repercussions across industry, healthcare, academia and personal health. With regards to disorders of sleep, reliable and scalable sleep monitoring is set to provide a better understanding of sleep disorder progression and severity. This could facilitate better and earlier diagnosis and decision-making for individual patients, including in instances where individuals need to be progressed to a new treatment. Digitisation may also be used in disease prevention and to provide lifestyle recommendations. Objective ubiquitous monitoring of sleep–wake cycles, combined with multi-modal data inputs reflecting an individual’s physical activity profiles, nutrition, all-day heart rate and genetic information will allow users to receive personalised feedback for health and well-being purposes and disease prevention. New technological advancements will allow for improved sleep coaching interventions that are aimed to improve sleep hygiene or provide with better recovery for example. Furthermore, data generated from these technologies could be used to help monitor the impact of pharmaceutical and post-operative interventions. Similarly, the accrued data gathered from clinical and epidemiological studies, as well as from commercial wearable devices, represents an unparalleled opportunity to deepen our understanding of the role of sleep in well-being and disease.

From the perspective of pharmaceutical companies, there are several benefits to the digitisation of sleep. Wearables offer the potential to deploy sleep monitoring at scale, in large populations that are required for late-phase clinical trials and can be used to provide better and earlier evidence of treatment efficacy in sleep disorders, thus facilitating the progression of promising candidates through trial phases. Further, there are implications for patient centricity. Across diseases, sleep is meaningful to patients and their health. It is therefore important to objectively assess sleep metrics such as sleep quality, WASO or time spent sleeping through quantitative measures, instead of relying on questionnaires. A summary of these metrics is provided in the Supplementary Fig. 1. Low-burden monitoring will facilitate sleep collection in trials and potentially help to increase trial participation and reduce attrition. Moreover, many metrics of sleep are strongly tied to the quality of life, thus, industry may welcome the use of these sensing technologies for post-market surveillance. The added knowledge of a potential positive impact of medicine on patients’ sleep quality may enable better reimbursement rates.

Ultimately, the digitisation of sleep could facilitate a truly personalised sleep monitoring experience, empowering people to improve their sleep^92^. However, the reproducibility and robustness of novel sleep monitoring and data analysis methods must be addressed prior to their use on large, longitudinal and multi-modal collaborative studies. The impact that these technologies can have on the management and understanding of sleep, as well as the treatment and prevention of sleep disorders, is set to be paradigm-changing. Industry, academic, public policy and clinical stakeholders should collaboratively enable this process of validation to take place, moving a step closer to truly personalised digital health.

In sum, digitisation of sleep and ubiquitous sleep monitoring will have important implications on the characterisation of sleep, diagnostics and therapeutics. Large-scale collection of objective, longitudinal sleep data through unobtrusive sleep sensing devices will facilitate epidemiological studies exploring the impact of sleep on health and disease. Furthermore, these applications will likely expand into sleep health, becoming increasingly accessible to individuals with the potential to empower and enable individuals to understand, manage and change their sleeping habits^36^.

## Supplementary information

Supplementary Material
