# Supplementary material for: The future of sleep health: a data-driven revolution in sleep science and medicine
Source: NPJ Digit Med. 2020 Mar 23;3:42. doi: 10.1038/s41746-020-0244-4 (PMC7089984; doi:10.1038/s41746-020-0244-4)
Supplement: Supplementary file 1 — Supplementary Material [file 41746_2020_244_MOESM1_ESM.pdf]

Supplementary Material
The Future of Sleep Health: A Data-Driven Revolution in Sleep Science and Medicine

Ignacio Perez-Pozuelo[1,2*], Bing Zhai[3], Joao Palotti[4,5], Raghvendra Mall[4*], Michaël Aupetit[4], Juan M Garcia-Gomez[6], Shahrad Taheri[7], Yu Guan[3]
Luis Fernandez-Luque[4]

[1]Department of Medicine, University of Cambridge, UK
[2]The Alan Turing Institute, UK
[3]Open Lab, University of Newcastle, UK
[4]Qatar Computing Research Institute, HBKU, Qatar
[5]CSAIL, Massachusetts Institute of Technology, USA
[6]BDSLab, Instituto Universitario de Tecnologias de la Informacion y Comunicaciones-ITACA, Universitat Politecnica de Valencia, Spain
[7]Department of Medicine and Clinical Research Core, Weill Cornell Medicine - Qatar, Qatar Foundation, Doha, Qatar
[*]Corresponding authors. E-mail: ip325@cam.ac.uk and rmall@hbku.edu.qa

Supplementary Note 1

Sleep Metrics

Beyond the sleep staging guidelines provided by AASM, there are several sleep metrics that are commonly used when assessing sleep-wake cycles. Sleep onset time is defined as the boundary that determines the transition between a period where the person is awake to when they are sleep. Similarly, the boundary between when a person is asleep and the transition to wake is known as the sleep awakening time. The following table introduces some of the most readily used sleep metrics based on these definitions.

Additionally, recently Phillips and colleagues described the Sleep Regularity Index (SRI) as "the likelihood that any two time-points, on a minute –to-minute basis, 24-hours apart were the same wake-sleep state, across all days[1]". So if we were to derive SRI using 30-second epochs on accelerometer data the SRI equation would be:

$$SRI = 100 + \frac{200}{M \cdot (N-1)} \sum_{j=1}^{M} \sum_{j=1}^{N-1} \sigma(S_{i,j}, S_{i+1,j})$$

Given N days of recorded divided into M (epoch=30s) daily epochs, suppose that $s_{i,j} = 1$ if sleep on day i and epoch j and 0 if they are awake.

New sleep metrics based on EEG data are currently being derived, in search of establishing reliable sleep EEG biomarkers that could be used to phenotype patients.[2,3] However, it is important that new sleep metrics also address the prevalence and representativeness of the data being used and account for it as well as the sampling and data-collection bias associated to sleep studies.

Supplementary Table 1

Supplementary Table 1: Conventional Sleep Metrics

Metric	Formula
Sleep Period Duration	‖ Sleep Awakening Time – Sleep Onset Time ‖
Sleep Period	[Sleep Onset Time, Sleep Awakening Time]
Wake After Sleep Onset (WASO)	$\sum_{n=onset}^{awake}$ ‖ Wakefulness ‖
Sleep Latency	[Preceding Sedentary Time, Sleep Onset Time]
Total Time in Bed (mins)	‖ Sleep Awakening Time – Preceding Sedentary Time ‖
Total Sleep Time (mins)	‖ Sleep Period Duration - WASO - Latency ‖
Sleep Efficiency (SE)	Total Sleep Time / Total Minutes in Bed

Supplementary Note 2

Classification Metrics

Understanding how well a specific method is performing at the classification task is of great importance for research, clinical, industry and lifestyle applications. While evaluating a method or model's accuracy can be insightful, it is not sufficient. In sleep-wake and sleep stage classification task not all errors are equal. Indeed, there are two categories of error: predicting a negative when the instance is positive and predictive a positive when the instance is negative. Moreover, there are two categories of good prediction: successful prediction is termed true and unsuccessful prediction is termed false. These four variants form a confusion matrix:

Supplementary Table 2

Supplementary Table 2: Confusion matrix: understanding false positives and false negatives in classification tasks

Data Class	Classified as pos	Classified as neg
pos	True positive (TP)	False negative (FN)
neg	False positive (FP)	True negative (TN)

From this, a variety of performance metrics can be derived, offering different perspectives on how the chosen method performs. Supplementary Table 3 introduces the most common metrics used for sleep-wake classification algorithm performance evaluation.

Supplementary Table 3

Supplementary Table 3: Assessing Classification Model Performance Through Metrics

Measure	Formula	Function/ focus
Accuracy	$\frac{TP+TN}{TP+FN+TN+FP}$	Overall effectiveness for the algorithm
Precision (Positive Predictive Value)	$\frac{TP}{TP+FP}$	Agreement between the data labels and positive labels given by the algorithm
Recall (Sensitivity)	$\frac{TP}{TP+FN}$	Effectiveness of the algorithm to identify positive labels
Specificity	$\frac{TN}{TN+FP}$	Effectiveness of the algorithm to identify negative labels
F1 Score	$\frac{2 \cdot Precision \cdot Recall}{Precision+Recall}$	Conveys the balance between the precision and recall of the algorithm
AUC	$\frac{1}{2}(\frac{TP}{TP+FN} + \frac{TN}{TN+FP})$	Algorithm's ability to avoid false classification

Supplementary Figure 1

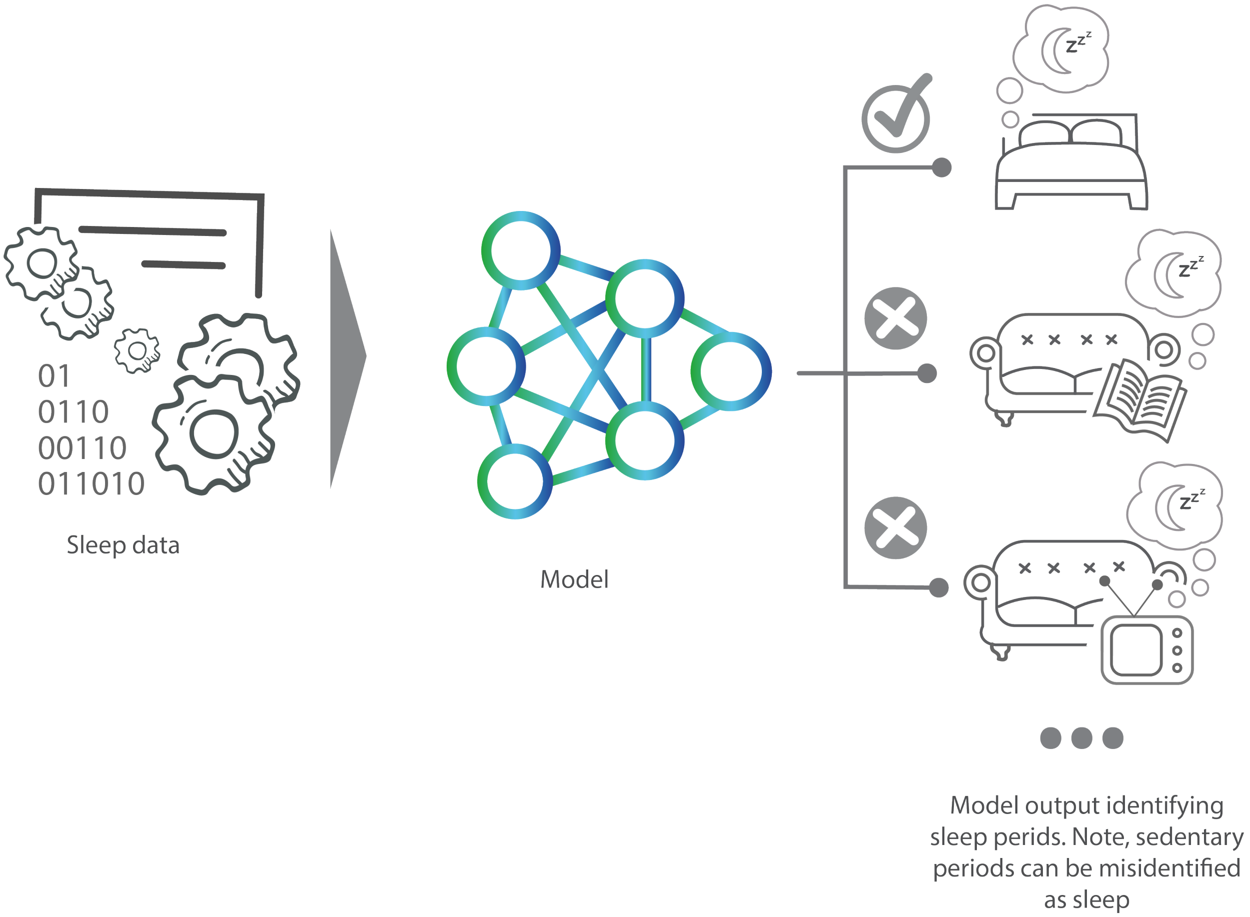

Supplementary Figure 1: The Importance of Classification Metrics: Precision, Sensitivity and Specificity for Sleep Classification are paramount to understand if the model is not only accurate, but also capable of discerning sleep from sedentary behaviours or other bed activities.

Supplementary Note 3

Performance Metrics on Sleep-Wake Classification

From a classification perspective, the correctness of the classification algorithms can be evaluated by the number of instances or events correctly recognised class examples (true positives), the number of instances or events that are correctly identified as not belonging to a certain class (true negatives), as well as the instances or events that are wrongly classified

as a given class (false positives) or were not recognised as a class (false negatives). Given these four metrics, we can compute what is known as a confusion matrix:

Based on the results obtained from the confusion matrix, there are several important metrics of performance that are derived to evaluate sleep classification algorithm performance. These are described in Supplementary Table 3.

Furthermore, when evaluating classification performance, other metric's are used depending on the characteristics of the data set. For instance, Cohen's Kappa is a metric that offers a comparison of the observed accuracy with respect to an expected accuracy (random chance, e.g. sleep-wake classification) and is defined as:

$$\kappa = \frac{Pr(a) - Pr(e)}{1 - Pr(e)}$$

Where Pr(a) is the observed accuracy and Pr(e) is the expected accuracy. Other commonly used metrics of classification accuracy are Hamming and hinge loss, the Matthews correlation coefficient or zero-one classification loss.

Figure 1 exemplifies the classification performance of a classifier. There are several difference instances in which the participant may be sleeping or engaging in other, sedentary, activities (like reading or watching TV). The task of the classifier is to correctly classify sleep as such in blue and in purple as other waking activities (like sedentary behaviors). The sensitivity (or recall), specificity and precision are presented for this example classification outcome.

Supplementary Note 4

Actigraphy Specific Sleep Metrics

Actigraphy data can be analyzed and studied to focus in either sleep or circadian rhythms. Traditional sleep metrics like the ones explored previously (Wake after sleep onset, total sleep time, etc) can be extracted from actigraphy data. Moreover, metrics related to circadian rhythms can also be derived. Some of the most common ones are interday stability (IS), intraday variability (IV), amplitude at rest (L5) and relative amplitude (RA).[4] These metrics provide information beyond that of traditional sleep metrics derived from actigraphy data.

For instance, intraday variability (IV) is a measure of sleep fragmentation and interday stability (IS) can be used to asses sleep regularity.

References

[1] Phillips, A. J. *et al.* Irregular sleep/wake patterns are associated with poorer academic performance and delayed circadian and sleep/wake timing. *Scientific reports* **7**, 3216 (2017).

[2] Penzel, T., Fietze, I. & Veauthier, C. The need for a reliable sleep eeg biomarker. *Journal of Clinical Sleep Medicine* **13**, 771–772 (2017).

[3] Levendowski, D. J. *et al.* The accuracy, night-to-night variability, and stability of frontopolar sleep electroencephalography biomarkers. *Journal of Clinical Sleep Medicine* **13**, 791–803 (2017).

[4] Forner-Cordero, A., Umemura, G. S., Furtado, F. & Gonçalves, B. d. S. B. Comparison of sleep quality assessed by actigraphy and questionnaires to healthy subjects. *Sleep Science* **11**, 141 (2018).
